# SARS-CoV-2 Delta–Omicron Recombinant Viruses, United States

**DOI:** 10.3201/eid2807.220526

**Published:** 2022-07

**Authors:** Kristine A. Lacek, Benjamin L. Rambo-Martin, Dhwani Batra, Xiao-yu Zheng, Norman Hassell, Hitoshi Sakaguchi, Thomas Peacock, Natalie Groves, Matthew Keller, Malania M. Wilson, Mili Sheth, Morgan L. Davis, Mark Borroughs, Jonathan Gerhart, Samuel S. Shepard, Peter W. Cook, Justin Lee, David E. Wentworth, John R. Barnes, Rebecca Kondor, Clinton R. Paden

**Affiliations:** Centers for Disease Control and Prevention, Atlanta, Georgia, USA (K.A. Lacek, B.L. Rambo-Martin, D. Batra, X. Zheng, N. Hassell, M. Keller, M.M. Wilson, M. Sheth, M.L. Davis, M. Burroughs, J. Gerhart, S.S. Shepard, P.W. Cook, J. Lee, D.E. Wentworth, J.R. Barnes, R. Kondor, C.R. Paden);; General Dynamics Information Technology, Inc., Atlanta (X. Zheng);; Retired, Yotsukaido City, Japan (H. Sakaguchi);; UK Health Security Agency, London, UK (T. Peacock, N. Groves);; Imperial College London, London (T. Peacock);; ASRT Incorporated, Smyrna, Georgia, USA (M.L. Davis, J. Gerhart)

**Keywords:** COVID-19, 2019 novel coronavirus disease, coronavirus disease, severe acute respiratory syndrome coronavirus 2, SARS-CoV-2, viruses, respiratory infections, zoonoses, Omicron, Delta, recombination, next-generation sequencing, bioinformatics, United States

## Abstract

To detect new and changing SARS-CoV-2 variants, we investigated candidate Delta–Omicron recombinant genomes from Centers for Disease Control and Prevention national genomic surveillance. Laboratory and bioinformatic investigations identified and validated 9 genetically related SARS-CoV-2 viruses with a hybrid Delta–Omicron spike protein.

Emerging variants of SARS-CoV-2 are characterized and monitored closely by national genomic surveillance. In addition to sequencing efforts from US public health, academic, and commercial laboratories, the Centers for Disease Control and Prevention (CDC) collects and sequences SARS-CoV-2 specimens from 64 partners across state, tribal, local, and territorial public health agencies through the National SARS-CoV-2 Strain Surveillance program (https://www.cdc.gov/coronavirus/2019-ncov/variants/cdc-role-surveillance.html) and funds SARS-CoV-2 sequencing through a nationwide network of commercial laboratory testing companies. To date, these efforts have contributed 1.8 million SARS-CoV-2 genomes from the United States to public repositories. The purpose of this genomic surveillance system is to detect and respond dynamically to new and changing SARS-CoV-2 variants (*1*).

Recombination is an evolutionary mechanism frequently observed in coronaviruses (*2*,*3*), and it can lead to rapid accumulation of mutations and heightened transmissibility (*4*). SARS-CoV-2 recombination events have also been found to arise disproportionately in the spike gene (Y. Turkahia et al., unpub. data, https://www.biorxiv.org/content/10.1101/2021.08.04.455157V1). Recombination between Alpha and Delta SARS-CoV-2 variants has been documented (*5*–*7*).

Given the divergence of the Delta and Omicron variant genomes, as well as the known immune-escape properties of Omicron (*8*,*9*), a Delta–Omicron recombinant strain could alter the landscape of vaccine and therapeutic effectiveness. In early 2022, viruses resulting from recombination between Delta and Omicron were reported, but further inspection indicated that these claims seemed to have resulted from laboratory artifact or co-infections (*10*). With this study, we identified candidate Delta–Omicron recombinant genomes from the CDC national genomic surveillance and attempted to rule out laboratory contamination or sequencing error.

## The Study

We identified 9 candidate recombinant sequences (Table) from CDC national genomic surveillance dataset made publicly available in GenBank and GISAID EpiCoV (https://www.gisaid.org). Using Bolotie, a rapid interclade recombination detection method (*3*), we identified these sequences as candidate recombinant genomes, having 1 parent in Delta (clade 21J) and 1 in Omicron (clade 21K). Bolotie describes a single breakpoint between nucleotide positions 22035 and 22577 (referenced to GenBank accession no. NC_045512.2); there are no differentiating mutations between clades 21J and 21K within this range. These sequences (EPI_ISL_8720194, EPI_ISL_9147438, EPI_ISL_9147935, EPI_ISL_8981459, EPI_ISL_8981824, EPI_ISL_9088187 [A. Bolze et al., unpub .data, https://www.medrxiv.org/content/medrxiv/early/2022/03/12/2022.03.09.22272113.full.pdf], EPI_ISL_8981712, EPI_ISL_10389339, EPI_ISL_10389336) contain hallmark mutation sets from both Omicron and Delta SARS-CoV-2 lineages, changing from Delta-associated substitutions to Omicron-associated substitutions between spike protein amino acids 158 and 339 (Appendix Figure 1, panel A). This breakpoint is distinct from the 2 clusters of apparent Delta–Omicron recombinants identified in the United Kingdom (https://github.com/cov-lineages/pango-designation/issues/422 and https://github.com/cov-lineages/pango-designation/issues/441), which have a breakpoint upstream of spike in the *ORF1ab* gene (Appendix Figure 1, panel A), and these samples show a singular breakpoint, unlike concurrently observed Delta–Omicron recombinants in France (P. Colson et al., unpub. data, https://www.medrxiv.org/content/10.1101/2022.03.03.22271812V1).

**Table:** Candidate recombinant samples, states, collection dates, and Bolotie outputs for the SARS-CoV-2 AY.119.2:BA.1.1 recombinant cluster, United States*

GISAID accession no.	State	GISAID virus name	Collection date	Bolotie results
EPI_ISL_8720194	TN	hCoV-19/USA/TN-CDC-ASC210559252/2021	2021 Dec 31	21J (Delta): 1-22032; 21K (Omicron): 22033-29903
EPI_ISL_9147438	NJ	hCoV-19/USA/NJ-CDC-IBX952397337138/2022	2022 Jan 4	21J (Delta): 1-22032; 21K (Omicron): 22033-29903
EPI_ISL_8981712	PA	hCoV-19/USA/PA-CDC-LC0473996/2022	2022 Jan 4	21J (Delta): 1-22032; 21K (Omicron): 22033-29903
EPI_ISL_8981824	PA	hCoV-19/USA/PA-CDC-LC0474055/2022	2022 Jan 4	21J (Delta): 1-22032; 21K (Omicron): 22033-29903
EPI_ISL_8981459	PA	hCoV-19/USA/PA-CDC-LC0474301/2022	2022 Jan 4	21J (Delta): 1-22032; 21K (Omicron): 22033-29903
EPI_ISL_9088187	MA	hCoV-19/USA/MA-CDC-STM-HZEBR92XC/2022	2022 Jan 12	21J (Delta): 1-22032; 21K (Omicron): 22033-29903
EPI_ISL_9147935	NJ	hCoV-19/USA/NJ-CDC-IBX640654818289/2022	2022 Jan 12	21J (Delta): 1-22032; 21K (Omicron): 22033-29903
EPI_ISL_10389336	NJ	hCoV-19/USA/NJ-CDC-ASC210553977/2022	2022 Feb 12	21J (Delta): 1-22032; 21K (Omicron): 22033-29903
EPI_ISL_10389339	NJ	hCoV-19/USA/NJ-CDC-ASC210553978/2022	2022 Feb 12	21J (Delta): 1-22032; 21K (Omicron): 22033-29903

*These 9 candidate recombinant viruses were identified by an exhaustive search of publicly available SARS-CoV-2 viral genomes with orf1ab:2855V,4176N,6248S and S:95I,142D,157-,346K,501Y mutations. hCoV-19/USA/PA-CDC-LC0474055/2022 and hCoV-19/USA/PA-CDC-LC0474301/2022 underwent resequencing at the Centers for Disease Control and Prevention. Bolotie (*3*) identified all 9 as recombinant genomes between Delta (clade 21J) and Omicron (clade 21K). Bolotie cannot determine the true breakpoint because of high sequence homology, but the same region is identified for all 9 sequences (nt position 22032 as referenced to GenBank accession no. NC_045512.2).

To rule out Delta and Omicron co-infection, laboratory contamination, and bioinformatic error, we examined the raw read data from the 9 candidate recombinants created from molecular loop and amplicon-based sequencing strategies. Two of these specimens were readily available from the original diagnostic laboratory, and extracted RNA was shipped to CDC for confirmatory sequencing. We used Illumina (https://www.illumina.com) and PacBio (https://www.pacb.com) sequencing of 2 whole-genome amplicon strategies, as well as spike-gene amplification followed by Oxford Nanopore (https://nanoporetech.com) sequencing (Appendix). All sequencing strategies yielded functionally identical consensus sequences compared with the corresponding original sequencing strategies.

Nextclade (*11*) classified the 9 whole genomes as 21K (Omicron/BA.1). We then split the genomes at position 22150 (within the predicted recombination site range). Nextclade classified the first 22150 base fragment as clade 21J (Delta) and the remainder as clade 21K (Omicron/BA.1). Pangolin version 3.1.20 (pangoLEARN 1.2.123, Scorpio 0.3.16, https://cov-lineages.org) assigned a lineage of none to the full-genome sequences. Pangolin classified the first 22150 base fragment of each recombinant as AY.43 (Delta), although the call was not supported by Scorpio. Inspection of this region revealed closer homology to AY.119.2 (Delta) sequences because of mutations orf1ab:A2855V and orf1ab:A6248S, which are common to AY.119 lineages, and orf1ab:K4176N, which is found in a subset of AY.119.2 (Delta) sequences. The remaining sequence fragment from nt 22151 to the 3′ end was classified by pangolin as BA.1.1 (Omicron). This observation has been documented in the PANGO-designations repository (https://github.com/cov-lineages/pango-designation/issues/439) and is under review for potential lineage assignment.

Detailed sequence analysis confirmed the 2 resequenced specimens as true recombinants and indicated no evidence of co-infection or contamination. Comparison with a representative AY.119.2 (Delta) specimen indicated characteristic Delta mutations (C21618G, C21846T, G21987A, and deletion 22029–22034) at >99% frequency (>600× coverage for Oxford Nanopore, >1,800× coverage for PacBio, >1000× coverage for Illumina) in the 5′ end of the recombinant (Appendix Figure 1, panel B). The 2 BA.1.1 (Omicron) deletions at the beginning of the spike gene (21765–21770 and 21987–21995) and the characteristic Omicron 9-base insertion after nt 22205 were not present in read data, consistent with a Delta origin for the 5′ end of the spike gene. After position 22577, the mutation profiles mirrored that of a representative BA.1.1 (Omicron) specimen (Appendix Figure 1, panel B). Analysis of individual Oxford Nanopore reads showed characteristic Delta mutations co-occurring with Omicron single-nucleotide variants on the same reads (sharing Delta 22029–22034 deletion and Omicron 22673 T>C; Appendix Figure 2). The translated spike protein is a hybrid, containing characteristic amino acids from both Delta and Omicron parents with a breakpoint between the N terminal domain and receptor-binding domain of spike S1 protein (Appendix Figure 1, panel A).

To visualize the parents of the recombinant genomes, we split all candidate recombinant genomes at position 22150, within the predicted breakpoint, and used Nextclade (*11*) to place each genome fragment (1–22150 and 22151 through the 3′ end) onto a reference tree. We visualized the 2 trees as a tanglegram tree with Auspice (*12*). Nucleotides 1–22150 clustered with clade 21J (Delta) sequences, and the remaining fragment of the genome clustered with 21K (Omicron/BA.1) (Appendix Figure 3).

## Conclusions

Our results provide evidence of a recombinant SARS-CoV-2 genome containing a hybrid spike protein derived from a Delta (AY.119.2)–Omicron (BA.1.1) recombination event. However, the ability to effectively identify and confirm additional recombinant viruses remains challenging because of the range of sequence quality available in the public domain. These limitations are a result of amplification inefficiency and consensus-calling algorithmic error, as well as cases of co-infection or potential sample contamination.

Comparative phenotypic characterization of virus isolates from the recombinant cluster was not possible because all specimens were chemically inactivated. In the spike protein, there are no additional amino acid substitutions within the receptor-binding domain compared with BA.1.1 (Omicron) lineage viruses. Recombinant viruses with this hybrid spike protein were detected over the course of 6 weeks, but the number of cases resulting from those viruses remains low. Most cases were identified within the mid-Atlantic region of the United States. However, epidemiologic linkage cannot be determined because CDC does not collect identifying information for these samples.

Systematic virus surveillance is essential for long-term monitoring of SARS-CoV-2 evolution. Given the potential public health consequences of new variants emerging from recombination, investigations involving laboratory and bioinformatic components, such as the one presented here, are critical for correctly identifying and tracking these viruses.

Appendix Supplemental methods and results for study of SARS-CoV-2 Delta–Omicron recombinant viruses, United States.
