# Supplementary material for: SARS-CoV-2 Delta–Omicron Recombinant Viruses, United States
Source: Emerg Infect Dis. 2022 Jul;28(7):1442–5. doi: 10.3201/eid2807.220526 (PMC9239886; doi:10.3201/eid2807.220526)
Supplement: Appendix — Supplemental methods and results for study of SARS-CoV-2 Delta–Omicron recombinant viruses, United States. [file 22-0526-Techapp-s1.pdf]

Article DOI: https://doi.org/10.3201/eid2807.220526

SARS-CoV-2 Delta–Omicron Recombinant Viruses, United States

Appendix

Supplemental Methods

Sample selection

We aligned candidate recombinant consensus sequences against representative sequences for Delta (AY.4, GISAID accession EPI_ISL_1758376) and Omicron (BA.1, GISAID accession EPI_ISL_7569944) and calculated Hamming distances, sequence quality, and locations of amino acid substitutions.

We also performed an exhaustive search of publicly available SARS-CoV-2 viral genomes with orf1ab:2855V,4176N,6248S and S:95I,142D,157-,346K,501Y variants to identify other potential AY.119:BA.1 recombinant genome sequences from publicly available sequence data in GISAID and NCBI's GenBank.

All samples identified were collected and sequenced as part of CDC's national genomic surveillance activity (*1*,*2*), also described here: https://www.cdc.gov/coronavirus/2019-ncov/variants/cdc-role-surveillance.html.

We found two complete samples matching the mutation profile for Delta (AY.119.2) until amino acid 339, then matched BA.1.1 (Omicron) through the end of the genome. Examination of raw read alignments showed no evidence of minor variant populations indicative of co-infection. We selected these two samples for additional sequencing via Pacific Biosciences, Oxford Nanopore Technologies, and Illumina platforms due to immediate availability, and high quality of the initial sequence data.

Sequencing Methods

We completed targeted Nanopore Spike gene sequencing with a custom primer set producing 2.2 and 2.5 kb amplicons overlapping across the S1-S2 cleavage site and extending beyond the coding region of the S-gene. Amplicons underwent native barcoding (LSK-109), and

GridION sequencing (Keller et al, in preparation). Live super-accurate basecalling was performed with guppy version 5.1.13 and reads were trimmed with BBDuk version 38.84. We assembled consensus genomes using the Iterative Refinement Meta-Assembler (IRMA) (*3*) on the CoV-s-gene configuration. Deletion thresholds required 10x depth with >50% frequency.

We prepared the samples using HiFiViral SARS-CoV-2 kit for sequencing on PacBio Sequel II and generated HiFi reads using PacBio SMRT Link v10.2.0.133424. Trimmed HiFi reads were assembled using IRMA with the CoV-pacbio configuration.

Finally, we performed 2x150 Illumina sequencing following library preparation with the IDT xGen SARS-CoV-2 Amplicon Panel (formerly Swift Normalase Amplicon Panel for SARS-CoV-2), down-sampled to 1 million reads per sample, trimmed primers with BBDuk, and assembled consensus genomes with IRMA's default CoV configuration.

We performed clade assignments using Nextclade version 1.13.2 (*4*) and assigned lineages using Pangolin version 3.1.20 (pangoLEARN 1.2.123, Scorpio 0.3.16) (*5*) for the whole genome, as well as upstream and downstream of the suspected recombination site independently.

Delta-Omicron Allele Profiles

We calculated the frequency of amino acid residues at each position along the genome within each PANGO lineage for all publicly available data. An allele was considered strongly "Delta" (leftmost orange in Appendix Figure 1) if the residue appeared in more than 50% of sequences in the Delta lineages (B.1.617.2 and AY sublineages), less than 50% of sequences in Omicron lineages (B.1.1.529, BA.1, BA.1.1, BA.2, and BA.3), and its frequency in Delta lineages was more than 20% greater than other lineages (non-Omicron, non-Delta). A residue was considered strongly Omicron (rightmost violet in Appendix Figure 1) if it appeared in more than 50% Omicron sequences, less than 50% of Delta sequences and its frequency in Omicron lineages was more than 20% greater than in other lineages. A residue that was otherwise Omicron, but greater than 20% of Delta sequences contain the residue, it is noted as "Omicron, minor Delta." Mutant residues that comprise >50% of both Omicron and Delta (common mutations to both) are colored grey. Residues more common in Omicron than Delta and comprising >0.25% Omicron are lighter hues of violet," and residues more common in Delta lineages than Omicron lineages and comprising >0.25% Delta are colored in lighter hues of orange. White colored alleles match the reference genome (Wuhan-Hu-1), and black colored

alleles are common to neither Omicron nor Delta genomes (although these may be common to a particular sublineage).

Recombination Analysis with Bolotie

Bolotie source code was obtained and installed (*6*). To build the conditional probability table for Bolotie, 226,173 SARS-CoV-2 genome sequences were selected and aligned to the NC_045512.2 genome. These sequences were published by CDC to GISAID before March 10, 2022, and each sequence contained less than 1% non-ATCG calls. Clade assignments for these sequences were obtained using Nextclade. Based on the phylogenetic relationships of Nextstrain clades, the clades were further binned into 10 large groups (1: 19B, 20A, 20B, 21B, 21D, 21H, 20C; 2: 20D, 21G, 20J, 21E; 3: 20G, 20H, 21C; 4: 21F; 5: 20I; 6: 21A; 7: 21I; 8: 21J (Delta); 9: 21K(Omicron); 10: 21L). A conditional probability table was built with this dataset and grouping using Bolotie default settings. The nine sequences of interest were then analyzed using this conditional probability table.

Phylogenomic Tree Generation

To obtain a reference sample set for the phylogenetic validation and comparison of recombinant samples, all available data (Internal CDC, GISAID, NCBI) was subset in the following fashion:

- Using Nextclade (v1.11.0) (*4*) clade assignments, five strain sequences from each non-Omicron and non-Delta clade were randomly sampled.
- 53 randomly sampled Delta variant strain sequences.
- 61 randomly sampled Omicron variant strain sequences.
- The Wuhan/Hu-1/2019 reference (to serve as a root sequence).

All reference samples were required to pass the following QC metrics: Nextclade “qc_overallstatus” = “good”, Nextclade “qc_missingdata_status” = “good”, Nextclade “privatenucmutations_totalprivatesubstitutions” < 10, Nextclade “privatenucmutations_totalreversionsubstitutions” < 1, 98% coverage of the spike protein coding frame, and no more than two ambiguous codes within the spike protein coding frame.

From this reference sequence set, two separate datasets were generated, by adding the “start” (positions 1-22150) and “end” (22150 onwards) portion of putative recombinant sample

genomes to the references. Two phylogenetic trees were generated using the Nextstrain bioinformatics toolkit (*7*) with custom "ncov" build profiles (https://github.com/nextstrain/ncov) for the "start" and "end" datasets. These trees were then visualized together in Nextstrain's auspice tree viewer with a tree "tangle-gram" to clearly indicate phylogenetic placement of the recombinant samples on the "start" and "end" dataset trees.

References

1. Lambrou AS, Shirk P, Steele MK, Paul P, Paden CR, Cadwell B, et al.; Strain Surveillance and Emerging Variants Bioinformatic Working Group; Strain Surveillance and Emerging Variants NS3 Working Group. Genomic surveillance for SARS-CoV-2 variants: predominance of the Delta (B.1.617.2) and Omicron (B.1.1.529) variants—United States, June 2021–January 2022. MMWR Morb Mortal Wkly Rep. 2022;71:206–11. PubMed https://doi.org/10.15585/mmwr.mm7106a4

2. Paul P, France AM, Aoki Y, Batra D, Biggerstaff M, Dugan V, et al. Genomic surveillance for SARS-CoV-2 variants circulating in the United States, December 2020–May 2021. MMWR Morb Mortal Wkly Rep. 2021;70:846–50. PubMed https://doi.org/10.15585/mmwr.mm7023a3

3. Shepard SS, Meno S, Bahl J, Wilson MM, Barnes J, Neuhaus E. Viral deep sequencing needs an adaptive approach: IRMA, the iterative refinement meta-assembler. BMC Genomics. 2016;17:708. PubMed https://doi.org/10.1186/s12864-016-3030-6

4. Aksamentov I, Roemer C, Hodcroft EB, Neher RA. Nextclade: clade assignment, mutation calling and quality control for viral genomes. J Open Source Softw. 2021;6:3773. https://doi.org/10.21105/joss.03773

5. O'Toole Á, Scher E, Underwood A, Jackson B, Hill V, McCrone JT, et al. Assignment of epidemiological lineages in an emerging pandemic using the pangolin tool. Virus Evol. 2021;7:b064. PubMed https://doi.org/10.1093/ve/veab064

6. Varabyou A, Pockrandt C, Salzberg SL, Pertea M. Rapid detection of inter-clade recombination in SARS-CoV-2 with Bolotie. Genetics. 2021;218:iyab074. PubMed https://doi.org/10.1093/genetics/iyab074

7. Hadfield J, Megill C, Bell SM, Huddleston J, Potter B, Callender C, et al. Nextstrain: real-time tracking of pathogen evolution. Bioinformatics. 2018;34:4121–3. PubMed https://doi.org/10.1093/bioinformatics/bty407

Appendix Table. Data availability

GISAID Accession	GISAID Virus Name	GenBank Accession	BioSample Accession
EPI_ISL_9088187	hCoV-19/USA/MA-CDC-STM-HZEBR92XC/2022	OM372355	SAMN25233756
EPI_ISL_10389336	hCoV-19/USA/NJ-CDC-ASC210553977/2022	OM835119	SAMN26256159
EPI_ISL_10389339	hCoV-19/USA/NJ-CDC-ASC210553978/2022	OM835120	SAMN26256158
EPI_ISL_9147935	hCoV-19/USA/NJ-CDC-IBX640654818289/2022	OM393452	SAMN25262455
EPI_ISL_9147438	hCoV-19/USA/NJ-CDC-IBX952397337138/2022	OM392955	SAMN25262195
EPI_ISL_8981712	hCoV-19/USA/PA-CDC-LC0473996/2022	OM344166	SAMN25170131
EPI_ISL_8981824	hCoV-19/USA/PA-CDC-LC0474055/2022	OM344238	SAMN25170117
EPI_ISL_8981459	hCoV-19/USA/PA-CDC-LC0474301/2022	OM344043	SAMN25170048
EPI_ISL_8720194	hCoV-19/USA/TN-CDC-ASC210559252/2021	OM272834	SAMN24974483

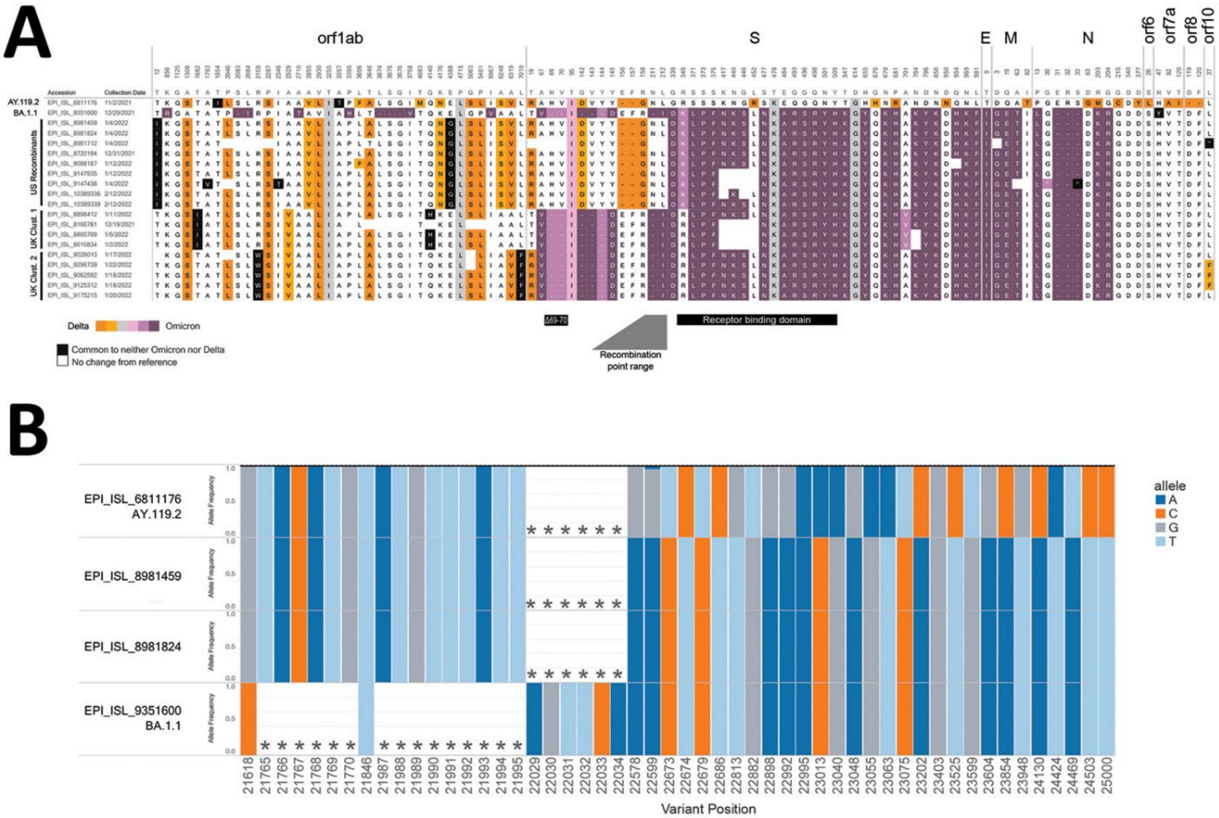

Appendix Figure 1. Composition of candidate recombinant SARS-CoV-2 genomes. A) Amino acid profiles of putative recombinants in the United States and United Kingdom. Delta variant–associated amino acid substitutions are shown in orange and Omicron variant–associated substitutions are shown in purple; hues correspond to the proportion of Omicron or Delta classified sequences that contain that substitution (Appendix, https://wwwnc.cdc.gov/EID/article/28/7/22-0526-App1.pdf). Grey boxes indicate

an amino acid substitution common to both Omicron and Delta sequences. White boxes indicate no change relative to Wuhan-Hu-1 virus, and black boxes denote substitutions that are not common to either Delta or Omicron sequences in aggregate. Distinct groups are shown; sequences from the United States appear to have recombination within the spike gene, and samples from 2 clusters from the United Kingdom show recombination upstream of the spike gene (UK cluster 1 represents https://github.com/cov-lineages/pango-designation/issues/445, UK cluster 2 represents https://github.com/cov-lineages/pango-designation/issues/441). The BA.1.1 (Omicron) deletion associated with spike-gene target failure (Δ69–70), the receptor-binding domain, and the range containing the recombination location are noted. B) Proportion of reads supporting each single-nucleotide variation and deletion around the recombination site from Illumina (IDT xGen amplicons) datasets generated at the Centers for Disease Control and Prevention. Shown are 2 recombinants (EPI_ISL_8981459, EPI_ISL_8981824), next to a representative AY.119.2 (Delta) genome ((EPI_ISL_6811176) and a representative BA.1.1 (Omicron) genome (EPI_ISL_9351600). Each bar shows the proportion of reads containing the given allele (colored by nucleotides A, C, T, and G) at each position for each sample. Asterisks denote deletions. Variants are relative to Wuhan-Hu-1 virus.

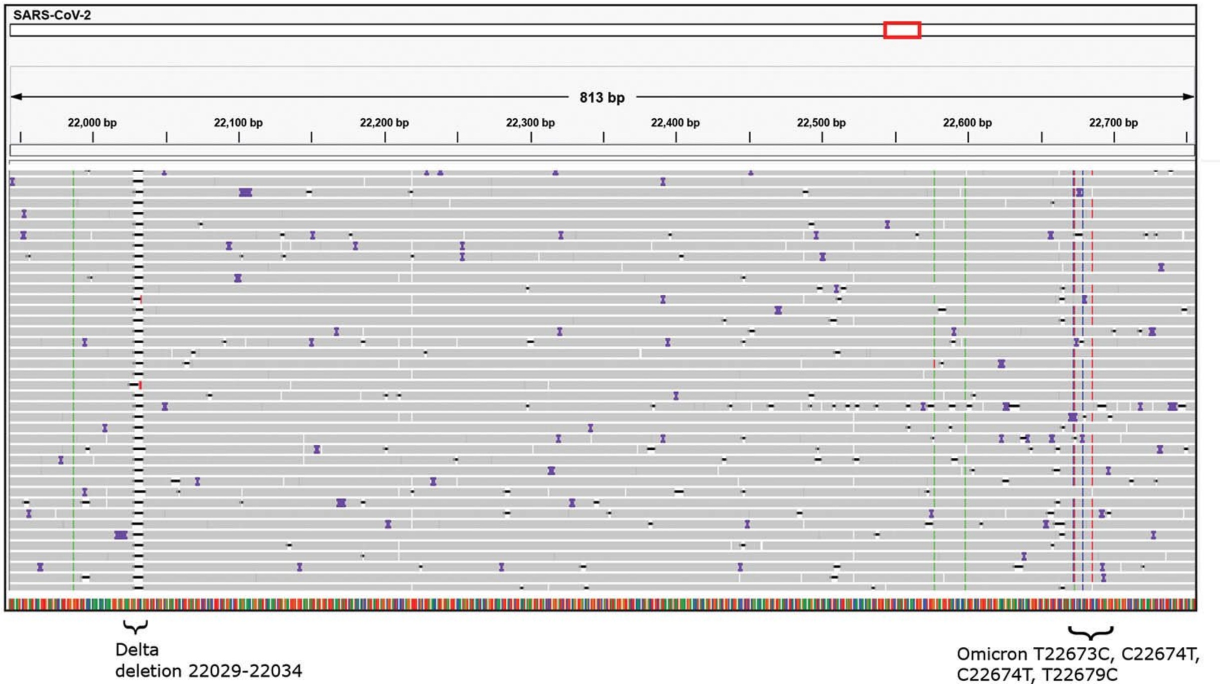

Appendix Figure 2. IGV Alignment of Nanopore long reads from one of the recombinant viruses demonstrating the presence of phased Delta 22029-22034 deletion and Omicron T22673C, C22674T, T22679C, C22686T SNVs originating from a single template. Green, red, and blue lines indicate a substitution with respect to Wuhan-Hu-1, while black bars indicate deletions and purple bars are spurious insertions.

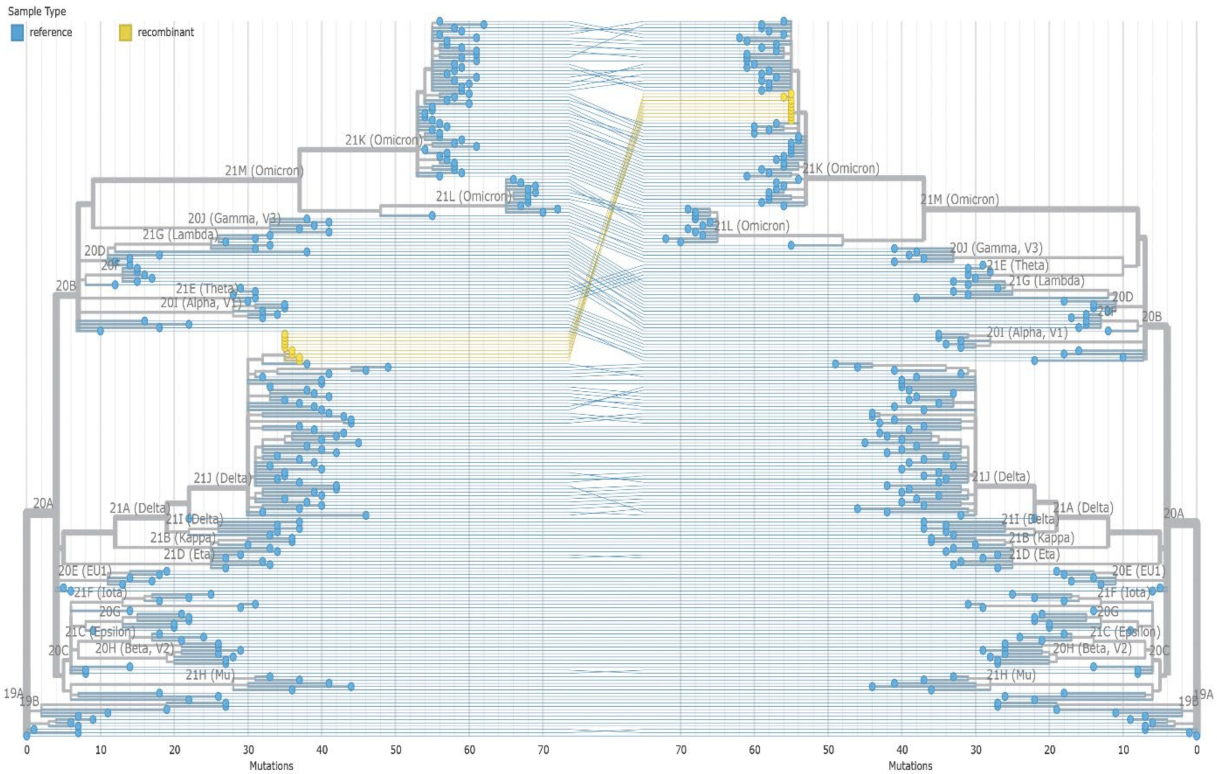

Appendix Figure 3. Tanglegram of candidate SARS-CoV-2 recombinants comparing the 5′ and 3′ sections of the genome. From a reference sequence set (Appendix, https://wwwnc.cdc.gov/EID/article/28/7/22-0526-App1.pdf), 2 separate datasets were generated by adding the start (positions 1–22150) and end (22150 on) portion of putative recombinant sample genomes to the references. Two phylogenetic trees (maximum-likelihood) were generated and visualized together in the Nextstrain Auspice tree viewer (*12*). The 9 putative recombinants are shown in yellow, and the connecting lines between trees pinpoint the corresponding sequences on each tree.
